# Cataract Grading in Pure Senile Cataracts: Pentacam versus LOCS III

**DOI:** 10.18502/jovr.v17i3.11570

**Published:** 2022-08-15

**Authors:** Mohammad Mirzaie, Erfan Bahremani, Nazli Taheri, Zhila Khamnian, Banafshe Kharrazi Ghadim

**Affiliations:** ^1^Nikoukari Eye Hospital, Department of Ophthalmology, School of Medicine, Tabriz University of Medical Sciences, Tabriz, Iran; ^2^Department of Community Medicine, Tabriz University of Medical Sciences, Tabriz, Iran

**Keywords:** Cataract, Lens Nucleus Densitometry, Lens Opacities Classification System III, Scheimpflug Lens Density, Pentacam

## Abstract

**Purpose:**

To determine the diagnostic accuracy of Pentacam Cataract Grading Scale (PCGS) versus the Lens Opacities Classification System (LOCS III) in scaling pure age-related cataract.

**Methods:**

Between April 2016 and May 2017, eyes of 281 patients were evaluated for grading of lens opacity. We used LOCS III and PCGS. Patients with pure age-related cataract with no previous history of eye surgery, eye trauma, or chronic systemic disease between 50 and 95 years of age were included. The examination of the patients was done, using slit lamp and LOCS III grading chart. The second examination was done a week later, using Oculus Pentacam. Next, we graded them using a PNS grading score. Spearman's rank correlation and a Bland-Altman Plot were implemented for analysis using MedCalc 14. 8.1. *P* < O.05 was considered as statistically significant.

**Results:**

Three hundred eyes were examined. Of them, 189 patients were male, and patients between 70 to 80 years old were the most common group. The correlation between grades of two methods was 0.47 (*P* < 0.001). Results of the Bland-Altman plot showed a moderate alignment between the two methods.

**Conclusion:**

The association between LOCSIII and PCGS is not so favorable, however, it is an economical and effective method to assess lens opacities is LOCSIII. PCGS can be used in early diagnosis. For a definitive diagnosis and appropriate therapeutic intervention, an ophthalmological examination is needed.

##  INTRODUCTION

Pure senile cataract is the most common cause of reversible age-related blindness worldwide.^[1,2]^ As the lens ages, chemical modification and proteolytic cleavage of lens proteins results in the formation of high-molecular-mass protein aggregates, thereby reducing transparency and increasing opacity.^[3,4]^ Cataract disease is a major public health issue around the world and poses a challenge especially to communities with rapidly ageing populations. Early diagnosis and appropriate therapeutic interventions are necessary to control the burden of this disease.^[5]^ Although the gold standard of therapy is the surgical removal of the opacified lens, there are varieties of objective and subjective methods to diagnose and confirm cataracts.^[6,7,8]^

One of the most commonly used subjective methods is the Lens Opacities Classification System (LOCS) III.^[4,9]^ This method is based on retro-illumination slit-lamp images and has been regarded as valid since 1993.^[10]^ This well-known system is used in the diagnosis and treatment of cataract-related patients.^[11,12]^

The LOCS III is a cost-effective valid method with high levels of reproducibility, but since it is subjective, it can be influenced by observer-related factors such as the experience level of the ophthalmologist, inter-observer and intra-observer bias, slit-lamp settings, the illumination method and amount and the necessarily subjective assessment of lens density and opacity.^[13]^ These limitations can lead to inconsistency in application over time and among different examiners,^[14]^ indicating an emerging need for a more reliable and reproducible method.^[15]^

Pentacam is an optical noncontact device of ophthalmologic examination that is programmed based on the principles of the Scheimpflug rotating camera system to capture images.^[16]^ Scheimpflug is able to provide a focused and sectional representation of the lens, making it superior to conventional slit-lamp imaging.^[17]^ It can also provide images. With a focus on a specific part of the lens, such as the nucleus.^[18]^

The currently available Pentacam rotating Scheimpflug system (Oculus, Inc., Wetzlar Germany) gathers information from 25,000 points across the eye by capturing as many as 50 “slices” in a single 180º sweep across the central axis of the lens within 2 sec.^[19]^ It provides a global evaluation of lens density and creates a precise single three-dimensional image of the anterior segment and lens.^[20]^ The technology of the system leads to a reduced artifact effect and improved lens densitometry because of the uniform four plane of focus.^[14]^ Pentacam Nucleus Staging (PNS) is a recently released software of Pentacam rotating Scheimpflug system that presents an accurate and precise measurement of lens density based on software capabilities such as generating a nuclear cataract grading score in five stages.^[10]^ PNS is a built-in lens densitometry software that provides an average and a maximal lens density with a cataract grading score from 0 to 5.^[19]^

Furthermore, PNS can calculate the mean value and uses Lens Densitometry Measurements by "Pentacam".^[21]^

Pentacam lens densitometry is influenced by varying levels of corneal opacities, incorrect photographic illumination, and insufficient pupil dilation. Film processing and analyzing are time consuming, and the use of the software is costly when compared to other alternatives.^[10]^

Accurate assessment of lens density and cataract grading is crucial for determining the optimal time for cataract surgery, choosing the appropriate surgical intervention, decreasing phaco time and phaco power, and minimizing peri- and post-operative complications.

In the present study, we tried to determine the association between the Pentacam Scheimpflug method and LOCS III for grading pure age-related cataracts and then find the correlation between two methods which are based on different imaging procedure.

##  METHODS 

In this prospective cross-sectional observer-masked study, 300 eyes of 281 age-related cataract patients were studied in a census manner. The study was conducted from April 2016 to May 2017 at Nikoukari Eye Hospital in Tabriz, Iran. The study protocol was approved by the Ethics Committee, Tabriz University of Medical Sciences and adhered to the guidelines of the Helsinki Declaration. An informed consent was obtained from all participants.

Patients referred to the hospital clinic during the study period and meeting the following inclusion criteria were recruited: age between 50 and 95 years, pure age-related nuclear cataract, and the desire to take part in the study. All patients with cortical or posterior subscapular cataracts, age-related cataract cornea or anterior segment pathologies, history of ocular trauma, prior history of ocular surgical intervention, or vitreoretinal disease were excluded. Patients with chronic systemic disorders affecting vision such as hypertension, diabetes mellitus, or thyroid disease were also excluded. The sample size was calculated based on Cochran's sample size formula 5 with a confidence level of 95% (a = 0.05), a power of 80%, and age-related cataract prevalence, resulting in a total sample of 300 eyes.

All participants underwent slit-lamp and fundus examination with a single experienced and trained ophthalmologist (MM) using a TOPCON Japan SL-3C Slit-lamp under full pupil dilation with Tropicamide I% ophthalmic drop (two drops; MydraxⓇ: Sinadarou, Tehran, Iran). Cataract grading was performed afterward by the same ophthalmologist according to LOCS III standards.^[4,13]^ LOCS III is measured with a slit-lamp examination composed of 16 pictures: six pictures for grading the color and opacity of lens nucleus, five retro-illumination pictures for cortical Garnet, and five retro-illumination pictures for posterior sub capsular cataract.^[22,23]^ The six groups of patients that were divided according to the nuclear opalescence were: Group 1, nuclear opalescence between 0.0 and 0.9; Group 2, between 2.0 and 2.9; Group 3, between 3.0 and 3.9; Group 4, between 4.0 and 4.9; Group 5, between 5.0 and 5.9; and Group 6, between 6.0 and 6.9.

After the slit-lamp examination, each eye was evaluated using a rotating Scheimpflug camera imaging device; an Oculus Pentacam (Oculus Inc. Germany) was used. Each participant underwent Pentacam examination after maximal pupil dilation by Tropicamide 1% ophthalmic drop (two drops; Mydrax Sinadarou Tehran, Iran). The patient was seated in front of Pentacam device like sitting in front of a slit lamp. The patient was asked to focus on a specific target and keep it fixed. The same device was used for all patients and was re-calibrated for each screening. Subsequently, the operator set the device on automatic mode to minimize operator-dependent variables. In this mode, detection of required settings with the corneal apex is done automatically, followed by an automated scanning image capturing 50 images. These images were taken from anterior segment, in about 2 sec. Using the three-dimensional plot of the anterior segment with each section running through the corneal vertex, we calculated the Jens density by using Pentacam software (PNS). Calculation was performed at 45º. The mean area was placed in the nucleus and measured 1.60 mm, 20% was the specified threshold value of the average nuclear density and nuclear cataract classification. Cataract grading score that the PNS software uses is 0 to 5.^[19]^ All examinations, whether slit-lamp or Pentacam were performed in the same room with minimal light sources to obtain reflex-free images and keep the environmental factor consistent for all patients.

Medcalc software version 14.8.1 was used for statistical analysis. Descriptive data is presented as median or frequency (percentage). Kolmogrov–Smimov's test was used for checking normality. Spearman's ranked correlation coefficient was calculated for determining the correlation between LOCS III and Pentacam Cataract Grading Scale (PCGS – PNS nuclear cataract grading score). A Bland-Altman plot was implemented for further studying the correlation between LOCS III and PCGS. The level of significance was considered to be *P* < 0.05.

**Table 1:** PNS Grading Score based on LOCS Ill classification.


**LOCS III**	**PNS Grading**	**Frequency**	**Percentage (%)**	**Median (PNS Grading Score)**	**Overall percentage**
1	0 1 2	2 7 2	18.2 63.6 18.2	1	0.66 0.33 0.66
2	0 1 2 3	6 94 60 5	3.6 57.0 36.4 3.0	1	2.00 31.33 20.00 1.66
3	1 2 3	26 62 21	23.9 56.9 19.3	2	8.66 20.66 7.00
4	1 2 3 5	2 3 8 1	14.3 21.4 57.1 7.1	3	0.66 1.00 2.6 0.33
5	4	1	100.0	4	0.33
	
	
PNS, Pentacam Nucleus Staging					

**Figure 1:**
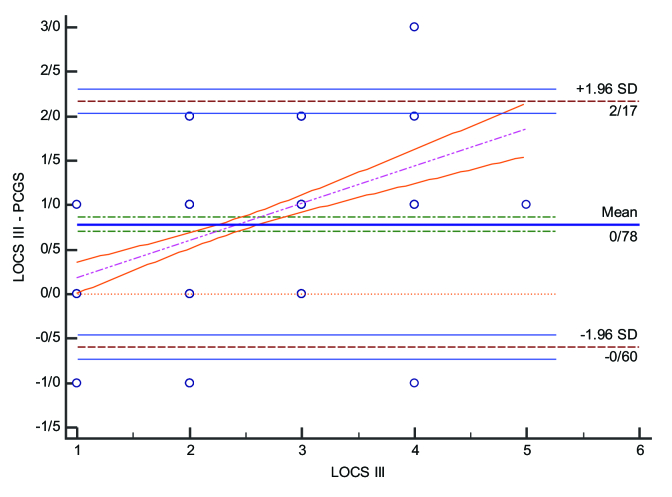
Bland-Altman Plot for LOCS III and PNS Grading Score (PCGS).

##  RESULTS

Three hundred eyes of 281 patients were recruited in the study; 19 patients were examined in both eyes. Ninety-two patients (32.7%) were female and 189 (67.3%) were male. The age range was 52 to 94 years, and the median age of participants was 75 years. Kolmogrov–Smirnov's test showed that the age distribution was near normal. Female patients were slightly older but the difference between two genders was not significant. Based on the results of the ophthalmologist's examinations, 11 eyes (3.7%) were Grade 1, 165 (55%) Grade 2, 109 (36.3%) Grade 3, 14 (4.7%) Grade 4, and just 1 was Grade 5 in the cataract classification according to LOCS III. No patient had a cataract grade above 5. The median grade was 2 and the mode was also 2. The Pentacam Oculus Automated examination for cataract grading revealed that 8 eyes (2.7%) were Grade 0, 129 (43%) Grade 1, 127 (42.3%) Grade 2, 34 (11.3%) Grade 3, 1 Grade 4, and one Grade 5 according to the PNS grading scale. Our data demonstrated that 99% of the patients were Grade 3 or lower based on the Pentacam examination and 99% were Grade 4 or lower in the LOCS III grading system. Table 1 shows the detailed results of Pentacam Grading scale with the LOCS III results.

The Spearman 's correlation coefficient was 0.47 between the ophthalmologist-graded cataract scaling using LOCS III and the PNS grading results via Pentacam examination based on Spearman's ranked correlation (*P* < 0.001). This level of association shows a moderate correlation between the results of these two methods. Because there were less than five eyes in some grades of PNS and in one grade of LOCS III, calculating the within-group coefficient was not possible based on statistical principles.

In our study, 95% of the eyes were graded at 3 or less on the LOCS III grading scale and 99% had a score of 4 or less on the PNS grading score. A Bland-Altman Plot for further assessing the association between measurements is shown below [Figure l]. This illustrates the correlation between LOCS III grading on the X-axis and the difference between the two methods on the Y-axis, showing that most cases staging with PNS have overlap with LOCS III about one standard deviation. In moderate cataract cases, the results were convergent, although in milder or higher grades of senile cataract, the two methods may reveal divergent results.

##  DISCUSSION

As a multifactorial disease, senile cataract is the most common type of cataract and a major cause of reversible nontraumatic visual impairment in all communities.^[5]^ Surgical intervention is the choice of treatment b replacing the opacified lens by an intact artificial lens.^[6]^ Many factors may predispose individuals to cataracts, such as trauma, certain drugs, ocular disorders, and many systemic diseases,^[24]^ however, most cataracts in senior citizens are caused by aging alone.^[25]^ Over 1.2 million surgeries are conducted for cure senile cataract in USA, annually.^[7]^ Definitive diagnosis and precise grading of senile cataracts are crucial in planning for treatment^[6]^ and there are distinctive objective and subjective tools for this purpose.

In this study, the association between the results of two methods, one subjective and one objective, was assessed. Our study found a moderate correlation between the LOCS III and the PNS grading scales. Magalhaes et al,^[10]^ in a cross-sectional study, evaluated 101 patients with clear cataracts for lens grading score. They have used LOCS III in this evaluation. Groups l to 5 could be noticed with Pentacam Lens Densitometry Program (PLDP) and the PNS mean value. However, there was a weak relationship between the PNS grading and the LOCS III classification. They further concluded that in a clinical setting, the mean value of nuclear density measured by PNS may be more accurate than the PNS score alone to evaluate the progress of nuclear opacification. They emphasized that the scores provided by Pentacam should be studied more.

Pei et al^[8]^ examined 180 eyes using both Scheimpflug Pentacam and LOCS III in 138 patients. They reported their results following a variety of variables they studied and found a positive linear correlation between LOCS III grading and the peak value of lens density measured using Pentacam in pure nuclear cataract patients. They concluded that the LOCS III criteria is a less expensive cataract grading system and provides data that are in concordance at a satisfactory level with results obtained using more expensive instruments. Therefore, LOCS III can be considered a reliable and economical method in estimating the severity of age-related nuclear cataracts in societies with fewer medical resources. Pan et al,^[23]^ in a cross-sectional study in 2015, evaluated the correlation using LOCS III against three other methods, including Pentacam, in senile cataract and found that the lens opacity measured according to LOCS III is strongly correlated with lens mean density, concluding that LOCS III is a cost-effective tool in diagnosing early cortical cataract. However, as they did not focus on pure nuclear cataracts, comparing results directly is less effective.

Gupta et al,^[13]^ during a longitudinal interventional research project, studied 100 eyes in 100 patients with pure age-related cataract for association between LOCS III and Scheimpflug maximum nuclear density grading scales with phacoemulsification parameters; all patients then underwent phacoemulsification and had permanent intraocular lenses implanted in their posterior chambers. They found positive linear correlation between the LOCS III grading and Scheimpflug maximum nuclear density. This correlation was stronger for the Pentacam imaging system with these phacoemulsification parameters.

Finally, Lim et al, based on a case–control design in 2015, evaluated Pentacam images measuring the lens density to recommend the most reasonable phaco time for each individual patient. Their investigation of 229 eyes with senile cataract revealed that the results of Pentacam imaging were associated with LOCS III classification, Nucleus Opacity (NO) and Nucleus Color (NC).^[19]^

Magalhaes et al reported a mild correlation between the PNS grading score and the LOCS III, and Lim et al found a stronger correlation. Our results were more consistent with the results of Magalhaes et al. However, as previous studies have analyzed the correlation based on Pearson's or Spearmen's correlation, our study implemented a Bland-Altman Plot to further investigate the association. Our results confirmed a mild-to-moderate correlation we had seen in Spearman's ranked correlation test.

### Limitations

The LOCS III method for classifying pure nuclear cataracts is an observer-dependent subjective tool that may cause measurement biases or inter-observer heterogeneity of results. On the other hand, Pentacam is an expensive method of examination because of the high cost of the Pentacam device.

### Conclusion 

Considering all available data, we suggest that LOCS III is the primary and remains the most useful and reliable grading tool for pure senile cataract diagnosis and treatment planning if done by an experienced, well-trained ophthalmologist. Considering all aspects of subjective and objective methods, the Oculus Pentacam is a valuable and accurate device for early diagnosis and cataract grading, but relying solely on Pentacam PNS grading is not wise. PNS needs to be considered together with other parameters that Pentacam software can measure and calculate.

Pentacam and its related software for cataracts might also be useful in primary healthcare for screening and early detection of senile cataract, however, an ophthalmologist is still needed to visit patients and examine eyes for cataract, since many factors may influence Pentacam cataract grading. Our study was performed on 300 eyes for comparing the results between these two methods. We collected a greater sample size compared to previous studies but lacked other parameters of the Pentacam examining tool. This study was aided by a single ophthalmologist for the LOCS III classification. Although the ophthalmologist was well-trained and highly experienced in cataract ophthalmologic examination, the use of a single doctor could cause observer bias. We would recommend that future studies should be designed based on a greater sample size and using two or more ophthalmologists for the LOCS III method, calculating their agreement coefficient, and utilizing the Pentacam system for further information.

##  Ethical Approval

The study protocol was registered with the Tabriz University of Medical Sciences and approved by the regional Committee of Ethics in Medical Research, and the protocol adhered to the guidelines of the Helsinki declaration. An informed consent was obtained from all participants.

##  Financial Support and Sponsorship 

The authors declare no funding or support for the present study.

##  Conflicts of interest

The authors declare that they have no conflict of interest.
